# Overexpression of *Jatropha Gibberellin 2-oxidase 6* (*JcGA2ox6*) Induces Dwarfism and Smaller Leaves, Flowers and Fruits in *Arabidopsis* and *Jatropha*

**DOI:** 10.3389/fpls.2017.02103

**Published:** 2017-12-12

**Authors:** Ying-Xiong Hu, Yan-Bin Tao, Zeng-Fu Xu

**Affiliations:** ^1^Key Laboratory of Tropical Plant Resources and Sustainable Use, Xishuangbanna Tropical Botanical Garden, Chinese Academy of Sciences, Mengla, China; ^2^College of Life Sciences, University of Chinese Academy of Sciences, Beijing, China

**Keywords:** *Jatropha*, *Arabidopsis*, gibberellin, GA2-oxidase, dwarf

## Abstract

Gibberellins (GAs) are plant hormones that play fundamental roles in plant growth and development. Gibberellin 2-oxidase (GA2ox) plays a direct role in determining the levels of bioactive GAs by catalyzing bioactive GAs or their immediate precursors to inactive forms. In this study, a *GA2ox* gene, designated *JcGA2ox6*, was isolated from *Jatropha curcas*. *JcGA2ox6* is expressed in all tissues of adult *Jatropha*, with the highest expression level in male flowers and the lowest expression level in young leaves. Overexpression of *JcGA2ox6* in *Arabidopsis* resulted in a typical dwarf phenotype, along with late flowering, smaller leaves and flowers, shorter siliques and smaller seeds. Similarly, when *JcGA2ox6* was overexpressed in *Jatropha*, the transgenic plants exhibited a dwarf phenotype with dark-green leaves and smaller inflorescences, flowers, fruits and seeds. However, the flowering time of *Jatropha* was not affected by overexpression of *JcGA2ox6*, unlike that in the transgenic *Arabidopsis*. Moreover, the number of flowers per inflorescence, the weight of 10 seeds and the seed oil content were significantly decreased in transgenic *Jatropha*. The results indicated that overexpression of *JcGA2ox6* had a great impact on the vegetative and reproductive growth of transgenic *Jatropha*. Furthermore, we found that the dwarf phenotype of transgenic *Jatropha* was caused by a decrease in endogenous bioactive GA_4_, which was correlated with the degree of dwarfism.

## Introduction

Physic nut (*Jatropha curcas* L.) is a perennial woody plant that belongs to the Euphorbiaceae family and is monoecious, with male and female flowers borne on the same inflorescence (Divakara et al., 2010). *Jatropha* seeds contain a high amount of oil and therefore represent a promising feedstock for renewable biodiesel production (King et al., 2009; Abdulla et al., 2011; Sato et al., 2011; Chakrabarti and Prasad, 2012) and bio-jet fuel (Li et al., 2010). However, the potential of *Jatropha* as a biofuel plant is limited by its low seed production, which results from excessive vegetative growth and erratic flowering and fruiting (Ghosh et al., 2010; Ye et al., 2014). Previous studies showed that soil application of the gibberellin (GA) biosynthesis inhibitor paclobutrazol (PAC) in *Jatropha* promoted the transition of shoot growth from the vegetative to reproductive phase, which resulted in significant increases in the numbers of inflorescence and infructescence per plant and, therefore, a higher seed yield (Ghosh et al., 2010; Song et al., 2013). Similar to *Jatropha*, PAC treatment reduced vegetative vigor and improved flowering in mangoes (Winston, 1992). In young macadamia trees, uniconazole, another type of GA biosynthesis inhibitor, was applied as a soil drench, resulting in flower initiation (Nagao et al., 1999). Reduced levels of endogenous GA have been correlated with flowering in citrus (Koshita et al., 1999) and lychee plants (Chen, 1990). These studies suggest that GA suppresses floral initiation in perennial woody plants, which is contrary to the role of GA in promoting flowering in most herbaceous plants such as *Arabidopsis* (Blazquez et al., 1998), maize (Evans and Poethig, 1995), and chrysanthemums (Dong et al., 2017). Therefore, it is valuable to identify the functional genes responsible for reducing the content of endogenous GA to promote the floral initiation of *Jatropha*.

Gibberellins are plant hormones that control diverse aspects of plant growth and development, such as seed germination, shoot elongation, leaf expansion, flower initiation and fruit development (Harberd et al., 1998; Fleet and Sun, 2005; Yamaguchi, 2008). Three major oxidase gene families of *GA 20-oxidase* (*GA20ox*), *GA 3-oxidase* (*GA3ox*) and *GA 2-oxidase* (*GA2ox*) participate in GA synthesis and degradation by a series of conversions from geranylgeranyl diphosphate (Hedden and Phillips, 2000). The *GA2oxs* are pivotal genes that reduce the endogenous bioactive GA content of plants with dwarfism (Lee and Zeevaart, 2005; Schwechheimer, 2008; Yamaguchi, 2008; Zhou et al., 2012). The functions of *GA2ox* genes have been characterized in various plant species. Overexpression of *AtGA2ox7* or *AtGA2ox8* in *Arabidopsis* decreased GA levels and flowering time was delayed in transgenic plants (Schomburg et al., 2003), and GA_4_ had been found to promote the transition from the vegetative to the reproductive phase (Eriksson et al., 2006; Yamaguchi et al., 2014). In rice, the transgenic plants carrying *Actin:OsGA2ox1* showed late flowering with low endogenous levels of GA_1_ (Sakamoto et al., 2003), and ectopic expression of *GA2ox6* can increase grain yield by 10–30% (Lo et al., 2017). However, in woody plant poplars, overexpression of *PtGA2ox* causes the dwarf trait and early flowering (Zawaski et al., 2011). These results led us to genetically inhibit vegetative growth and promote reproductive growth in *Jatropha* by overexpressing *Jatropha GA2ox* genes.

Based on a previous study, there are five GA2-oxidase homologue genes in *Jatropha* (Gao et al., 2015), designated as *JcGA2ox2* (GenBank accession No. KDP37976), *JcGA2ox4* (GenBank accession No. KDP27967), *JcGA2ox6* (GenBank accession No. KDP28294), *JcGA2ox7* (GenBank accession No. KDP39055), and *JcGA2ox8* (GenBank accession No. KDP30016). In this study, we isolated *JcGA2ox6* and analyzed its function in transgenic *Arabidopsis* and *Jatropha*, and we found that overexpression of *JcGA2ox6* had a significant impact on plant growth and development. The transgenic plants exhibited a typical dwarf phenotype with darker green leaves and smaller reproductive organs.

## Materials and Methods

### Plant Materials and Growth Conditions

*Jatropha curcas* plants cultivated in Xishuangbanna, Yunnan Province, China, were used in this study as described previously (Pan and Xu, 2011). The *Arabidopsis thaliana* ecotype Columbia (Col-0) and the transgenic lines were grown in plant growth chambers at 22 ± 2°C under long-day (LD, 16 h light/8 h dark) or short-day (SD, 8 h light/16 h dark) conditions. Phenotype analysis was performed on homozygous (T3) *Arabidopsis* plants and heterozygous (T0) *Jatropha* plants.

### Cloning of *JcGA2ox6* cDNA

Total RNA was extracted from the leaves of flowering *Jatropha* plants using the protocol described by Ding et al. (2008). First-strand cDNA was synthesized using M-MLV reverse transcriptase from TAKARA (Dalian, China) according to the manufacturer’s instructions. A full-length *JcGA2ox6* CDS was amplified by PCR using the primers XA579 and XA580 (Supplementary Table 1), which introduced *Bam*HI and *Sal*I restriction sites at the ends of the *JcGA2ox6* CDS fragment, respectively. The PCR products were subsequently cloned into the pGEM-T vector (Promega Corporation, Madison, WI, United States) and sequenced. All primers used in this research are listed in Supplementary Table 1.

### Sequences and Phylogenetic Analyses of JcGA2ox6

The JcGA2ox6 amino acid sequence was deduced according to the coding sequence (GenBank accession No. KDP28294). Related sequences were identified through a BLAST search^1^. To determine the amino acid identities, the alignment results were subjected to pairwise comparisons using DNAMAN 6.0. A phylogenetic tree was constructed based on the protein sequences with MEGA 5.0.^2^ A neighbor-joining phylogenetic tree was generated with MEGA 5.0 using the Poisson model, with gamma-distributed rates and 1,000 bootstrap replicates.

### Construction of Plant Expression Vectors and Transformation of *Arabidopsis* and *Jatropha*

To construct the plant overexpression vector *35S:JcGA2ox6*, the *JcGA2ox6* sequence was excised from the pGEM-T vector (Promega, Madison, WI, United States) using the restriction enzymes *Bam*HI and *Sal*I. Then, *JcGA2ox6* was cloned into the pOCA30 vector containing the *CaMV35S* promoter and the *35S* enhancer (Chen and Chen, 2002). The *JcUEP* promoter (Tao et al., 2015) was obtained by PCR from the *Jatropha* genomic DNA using the primers XB348 and XB349 (Supplementary Table 1), which introduced *Hind*III and *Sac*I restriction sites, respectively. The PCR products were cloned into pGEM-T and sequenced. To construct the *JcUEP:JcGA2ox6* plasmid, the *CaMV35S* promoter of the *35S:JcGA2ox6* vector was replaced with the *JcUEP* promoter using the restriction enzymes *Hind*III and *Sac*I.

Transformation of *Arabidopsis* with the *Agrobacterium* strain EHA105 carrying the *35S:JcGA2ox6* construct was performed using the floral dip method (Clough and Bent, 1998). Transformation of *Jatropha* with the *Agrobacterium* strain EHA105 carrying the *35S:JcGA2ox6* and *JcUEP:JcGA2ox6* constructs was performed according to the protocol described by Pan et al. (2010) and Fu et al. (2015).

### Expression Analysis by Quantitative RT-PCR (qRT-PCR)

Total RNA was extracted from frozen *Jatropha* tissues as described by Ding et al. (2008). *Arabidopsis* total RNA was extracted from frozen tissues using TRIzol reagent (Transgene, China). First-strand cDNA was synthesized using the PrimeScript^®^ RT Reagent Kit with gDNA Eraser (TAKARA, Dalian, China). qRT-PCR was performed using SYBR^®^ Premix Ex Taq^TM^ II (TAKARA) on a Roche 480 Real-Time PCR Detection System (Roche Diagnostics). qRT-PCR was performed using three independent biological replicates and three technical replicates for each sample. Data were analyzed using the 2^-ΔΔCT^ method as described by Livak and Schmittgen (2001). The transcript levels of specific genes were normalized using *Jatropha JcActin1* or *Arabidopsis AtActin2*. The primers used for qRT-PCR are listed in Supplementary Table 1.

### Chlorophyll Content Measurement

Three 1-cm^2^ leaf segments were removed from mature leaves of wild-type (WT) and transgenic *Jatropha* using a hole punch. The total chlorophyll, chlorophyll a and chlorophyll b contents were measured following the protocol described by Arnon (1949). Each measurement was repeated three times.

### Quantification of Endogenous GAs

The WT and T1 transgenic *Jatropha* (lines L43 and L27) were grown in soil for 6 weeks in a growth chamber at 28°C under a 16 h light/8 h dark photoperiod (**Figure 8A**). Their young stems (from shoot tip to node 2) (**Figure 8B**) were collected for GA quantification. The GA contents were determined by the Wuhan Greensword Creation Technology Company, and the analysis was performed as described previously (Chen et al., 2012). Three independent biological replicates and three technical replicates were measured for each sample.

## Results

### Cloning and Sequence Analysis of *JcGA2ox6*

A combined reverse transcriptase-polymerase chain reaction (RT-PCR) strategy was used to isolate *JcGA2ox6* cDNA from *Jatropha*. The *JcGA2ox6* coding sequence (CDS) (GenBank accession no. KDP28294) comprises 1002 bp and encodes a 333-amino-acid protein. A multiple alignment was performed using the JcGA2ox6 sequence and the sequences of GA2ox homologs from other species (Supplementary Figure 1A). JcGA2ox6 showed 84, 74, 71, and 57% sequence identity with *Ricinus communis* GA2ox5 (RcGA2ox5), *Populus trichocarpa* GA2ox2 (PtGA2ox2), *Vitis vinifera* GA2ox5 (VvGA2ox5) and *Arabidopsis thaliana* GA2ox6 (AtGA2ox6), respectively.

To better characterize the *JcGA2ox* genes within the large *GA2ox* family, a phylogenetic analysis was performed with amino acid sequences of the GA2oxs in some plant species (Supplementary Figure 1B). The analysis showed that JcGA2ox2, JcGA2ox4, and JcGA2ox6 have the highest identity with RcGA2ox2, RcGA2ox4, and RcGA2ox5 protein from *Ricinus communis*, respectively, and that they clustered with the C_19_-GA2ox group of *Arabidopsis*. This result suggests that all of them encode this type of enzyme. In contrast, JcGA2ox7 and JcGA2ox8 clustered with the C_20_-GA2oxs group of *Arabidopsis*.

### Expression Pattern of *JcGA2ox6* in *Jatropha*

**FIGURE 1:**
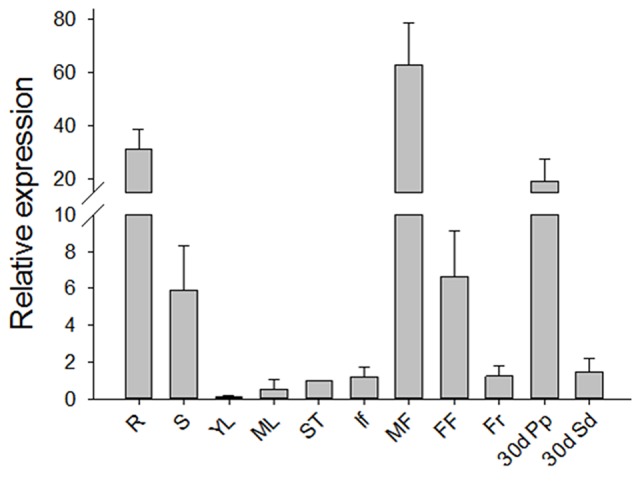
Expression profile of *JcGA2ox6* in three-year-old adult *Jatropha*. The qRT-PCR results were obtained from three independent biological replicates and three technical replicates for each sample. R, roots; S, stems; YL, young leaves; ML, mature leaves; ST, shoot tips; If, inflorescences; MF, male flowers; FF, female flowers; Fr, fruits at 10 days after pollination (DAP); 30d Pp, pericarps at 30 DAP; 30d Sd, seeds at 30 DAP. The levels of the detected amplicons were normalized using the amplified products of the *JcActin1*. The mRNA level in the shoot tips was set as the standard, with a value of 1. The values are presented as the means ± standard deviation.

To investigate the expression pattern of *JcGA2ox6* in *Jatropha*, we performed a qRT-PCR analysis with the total RNAs extracted from various tissues of adult plants, including the roots, stems, young and mature leaves, shoot tips, inflorescences, male and female flowers, fruits at 10 days after pollination (DAP), pericarps at 30 DAP and seeds at 30 DAP. The expression profile showed that *JcGA2ox6* was almost constitutively expressed in adult *Jatropha* (**Figure 1**). The primary expression levels were detected in the roots, stems, flowers and pericarps at 30 DAP, with the highest level found in male flowers. Very low expression levels were detected in the mature leaves, shoot tips, inflorescences, fruits and seeds at 30 DAP, especially the young leaves.

### Overexpression of *JcGA2ox6* in *Arabidopsis* Caused the Dwarf Phenotype with Late Flowering, Smaller Flowers, Shorter Siliques and Smaller Seeds

**FIGURE 2:**
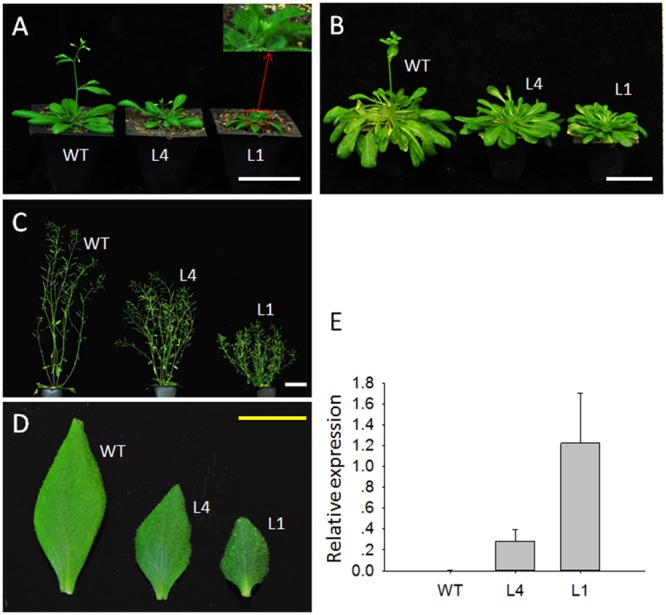
Ectopic expression of *JcGA2ox6* causes the dwarf phenotype with small leaves and late flowering in transgenic *Arabidopsis*. **(A)** Thirty-five-day-old plants grown under LD conditions. **(B)** One-hundred-twenty-five-day-old plants grown under SD conditions. **(C)** Seventy-five-day-old plants grown under LD conditions. **(D)** The first cauline leaves of WT and transgenic *Arabidopsis* plants under LD conditions. **(E)** qRT-PCR analysis of *JcGA2ox6* in WT and transgenic *Arabidopsis* plants. The levels of the detected amplicons were normalized using the amplified products of *AtActin2*. The values are presented as the means ± standard deviation. WT, wild type; L4 and L1 represent independent *35S:JcGA2ox6* transgenic *Arabidopsis* lines. White bars = 5 cm, yellow bar = 1 cm.

To determine the roles of *JcGA2ox6* in plant growth and development, *35S:JcGA2ox6* was transformed into *Arabidopsis* for preliminary analysis. WT *Arabidopsis* under the same growth conditions was used as a control. Transgenic plants were confirmed by qRT-PCR analysis of *JcGA2ox6* expression using rosette leaves. More than twenty independent T1 transgenic lines were generated with the *35S:JcGA2ox6* construct. Transgenic plants showed high *JcGA2ox6* expression levels (**Figure 2E**). Under LD conditions, most transgenic lines showed a dwarf phenotype with late flowering.

**FIGURE 3:**
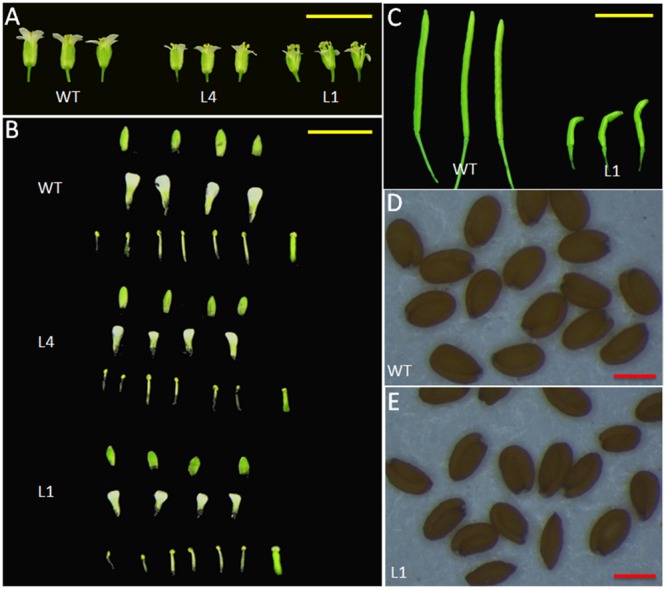
Morphological changes in the reproductive organs of *35S:JcGA2ox6* transgenic *Arabidopsis*. **(A)** Flowers from WT and transgenic L4 and L1 lines. **(B)** Anatomy of the flowers. **(C)** Siliques from the WT and transgenic line L1. **(D,E)** Seeds from the WT and transgenic line L1. Yellow bars = 5 mm, red bars = 0.3 mm.

We selected two independent homozygous lines (L4 and L1) in the T3 generation to examine the phenotypes. Compared with the WT plants, *Arabidopsis* overexpressing *JcGA2ox6* were 12.1–23 cm shorter (**Figure 2C** and **Table 1**) and produced smaller rosette and cauline leaves (**Figures 2A,D**) under LD conditions. Furthermore, the transgenic lines bolted later under both LD and SD conditions, but there was no significant difference in rosette leaf number (**Figures 2A,B** and **Tables 1, 2**). The phenotypes of the transgenic *Arabidopsis* were similar to that of the GA-deficient mutant *ga1–3* (Koornneef and Vanderveen, 1980), which suggested that overexpression of *JcGA2ox6* reduced the endogenous GA levels. Furthermore, flower development was also affected, as smaller sepals, petals, stamens and pistils were noted (**Figures 3A,B**). However, the transgenic plants were fertile, producing shorter siliques and smaller seeds (**Figures 3C–E**).

**Table 1:** Overexpression of *JcGA2ox6* caused dwarf phenotype with late flowering in transgenic *Arabidopsis* under LD conditions.

Lines	*N*	Rosette leaves	Flower bud formation time (Day)	Height (cm)
WT	16	12.63 ± 0.72	25.19 ± 1.28	37.58 ± 2.18
L4	16	12.94 ± 0.68	29.06 ± 1.88^∗∗^	25.44 ± 1.72^∗∗^
L1	16	12.68 ± 0.60	30.38 ± 2.28^∗∗^	14.63 ± 2.65^∗∗^

*WT plants and two independent *JcGA2ox6*-overexpressing lines (L4 and L1) grown under LD conditions (16 h light/8 h dark) were subjected to the analysis of rosette leaves, flowering times and heights. N = plant number. The values are presented as the means ± standard deviation. ^∗∗^Significantly different from the control at the 1% level.*

**Table 2:** Overexpression of *JcGA2ox6* causes late flowering in transgenic *Arabidopsis* under SD conditions.

Lines	*N*	Rosette leaves	Flower bud formation time (Day)
WT	15	57.40 ± 3.94	123.07 ± 4.80
L4	12	58.83 ± 5.72	132.25 ± 3.52^∗∗^
L1	12	60.33 ± 5.48	137.67 ± 4.10^∗∗^

*WT plants and two independent *JcGA2ox6*-overexpressing lines (L4 and L1) grown under SD conditions (8 h light/16 h dark) were subjected to the analysis of rosette leaves and flowering times. N = plant number. The values are presented as the means ± standard deviation. ^∗∗^Significantly different from the control at the 1% level.*

### Overexpression of *JcGA2ox6* in *Jatropha* Caused a Dwarf Phenotype with Small Dark-Green Leaves

To further test whether *JcGA2ox6* behaves accordingly in *Jatropha*, the transgenic *Jatropha* overexpressing *JcGA2ox6* was generated. Transgenic shoots overexpressing *JcGA2ox6* under the control of the *CaMV35S* promoter showed abnormal phenotypes of severely crimpled leaves and no obvious stems (Supplementary Figure 2). Moreover, these shoots were unable to generate roots. Therefore, we grafted these shoots onto WT rootstocks, but they hardly grew. These observations prompted us to replace the *CaMV35S* promoter with a weaker, *JcUEP* promoter, which is an alternative to the *CaMV35S* promoter for driving constitutive overexpression of transgenes in *Jatropha* (Tao et al., 2015). We successfully generated more than 30 transgenic *Jatropha* lines carrying the *JcUEP:JcGA2ox6* transgene.

**FIGURE 4:**
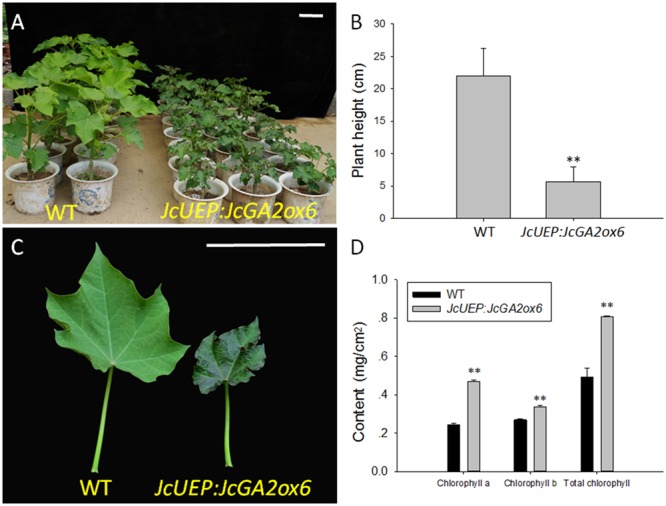
Phenotypic analysis of *JcUEP:JcGA2ox6* transgenic *Jatropha* plants grown in pots for 2 months. **(A,B)** A height comparison between WT and transgenic *Jatropha*. **(C)** The mature leaves from the WT and transgenic *Jatropha* plants. **(D)** Chlorophyll a, chlorophyll b and total chlorophyll contents of the mature leaves. The values are presented as the means ± standard deviation. Bars = 10 cm. ^∗∗^Significantly different from the control at the 1% level.

**FIGURE 5:**
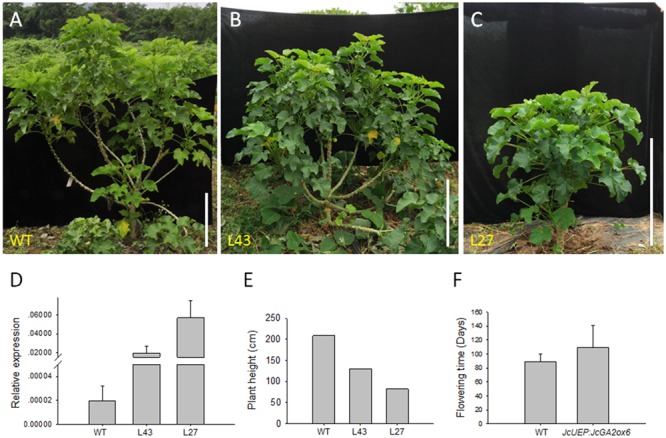
Dwarf phenotype of *JcUEP:JcGA2ox6* transgenic *Jatropha* grown in the field for 5 months. **(A)** WT. **(B,C)** Transgenic *Jatropha* (L43 and L27). **(D)** qRT-PCR analysis of *JcGA2ox6* in the WT and transgenic *Jatropha* plants. The levels of the detected amplicons were normalized using the amplified products of *JcActin1*. The values are presented as the means ± standard deviation. **(E)** Comparison of the heights of the WT and transgenic *Jatropha* plants. **(F)** Comparison of the flowering time of the WT and transgenic *Jatropha* plants. The values are presented as the means ± standard deviation. Bars = 50 cm.

When all plants grew in pots for 2 months, the *JcUEP:JcGA2ox6* transgenic plants exhibited a dwarf phenotype with small dark-green leaves (**Figure 4A**). The average height of the transgenic *Jatropha* plants was decreased by 74% compared with that of the WT plants (**Figure 4B**). Since the leaves of transgenic plants turned dark green, the mature leaves (**Figure 4C**) were collected to measure the chlorophyll content. The average chlorophyll a, chlorophyll b and total chlorophyll contents in the transgenic plants were 1.9-, 1.3-, and 1.6-fold higher than those of the WT plants, respectively (**Figure 4D**). This result is consistent with studies in for example transgenic potato (Jackson and Prat, 1996; Martínez-García et al., 2001) and soybean (Suo et al., 2012), which showed that the chlorophyll concentration was inversely correlated with GA_1_ or GA_1_ and GA_4_ contents. In *Solanum* species, overexpression of a gibberellin 2-oxidase gene from *Phaseolus coccineus* L. produced darker green leaves with higher concentrations of chlorophylls (Dijkstra et al., 2008). After 5 months of plant growth in the field, the transgenic plants were still dwarfed (**Figures 5A–C**). In addition, shoot branching was not affected by this transgene. The numbers of branches in WT and transgenic *Jatropha* were almost the same. We examined the phenotypes of two independent transgenic *Jatropha* lines L43 and L27, which exhibited intermediate and high expression levels of *JcGA2ox6*, respectively (**Figure 5D**). Accordingly, the heights (from the soil to the highest part of the main stem) of L43 and L27 lines were decreased by 37 and 60%, respectively, compared with that of the WT plant (**Figure 5E**). The retarded vegetative growth was similar to that of transgenic *Arabidopsis* (**Figure 2C** and **Table 1**).

### Overexpression of *JcGA2ox6* in *Jatropha* Affected Flower, Fruit and Seed Development

**FIGURE 6:**
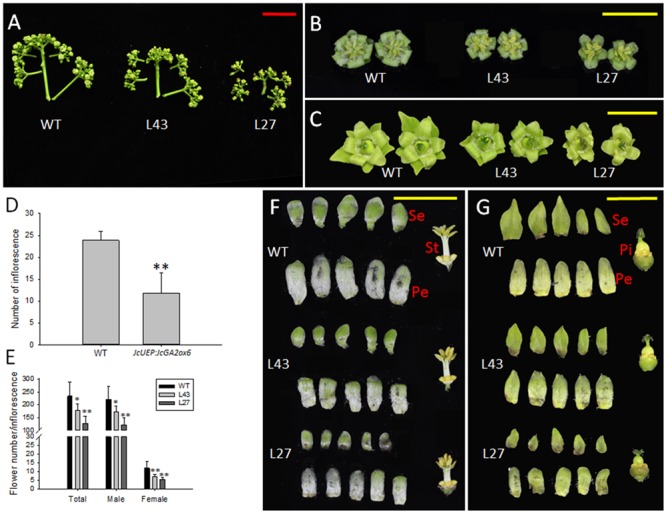
Inflorescence and flower development in *JcUEP:JcGA2ox6* transgenic *Jatropha*. **(A)** Inflorescences of WT and transgenic *Jatropha* (L43 and 27). **(B)** Male flowers of WT and transgenic *Jatropha* (L43 and 27). **(C)** Female flowers of WT and transgenic *Jatropha* (L43 and 27). **(D)** The inflorescence numbers in WT and transgenic *Jatropha*. Six WT plants and eight transgenic plants were analyzed. **(E)** The flower numbers per inflorescence in WT and transgenic *Jatropha* plants. The values are presented as the means ± standard deviation; ^∗^significantly different from the control at the 5% level, ^∗∗^significantly different from the control at the 1% level. **(F)** Anatomy of male flowers from WT and transgenic *Jatropha* (L43 and 27). **(G)** Anatomy of female flowers from WT and transgenic *Jatropha* (L43 and 27). Se, sepals; Pe, petals; St, stamens; Pi, pistils. Red bar = 5 cm, yellow bars = 1 cm.

When WT and transgenic plants were grown in the field for approximately 3 months, inflorescence buds emerged in both plants (**Figure 5F**), suggesting that the flowering time of *Jatropha* was not affected by overexpression of *JcGA2ox6*, unlike the late-flowering noted in transgenic *Arabidopsis*. The inflorescence numbers in the transgenic lines, contrary to our expectation, were lower than those in the WT plants (**Figure 6D**). Additionally, each inflorescence produced fewer male and female flowers (**Figure 6E**). Moreover, the inflorescences size was reduced in the transgenic *Jatropha* lines (**Figure 6A**) because of the shorter inflorescence stalks (Supplementary Figure 3A) and smaller male and female flowers (**Figures 6B,C** and Supplementary Figures 3B–E). In addition, similar to transgenic *Arabidopsis* (**Figure 3B**), the floral organs remained intact in transgenic *Jatropha* (**Figures 6F,G**) and plants were fertile.

**FIGURE 7:**
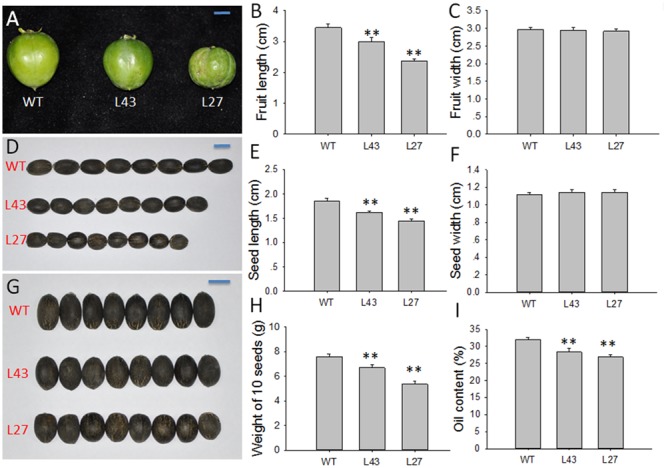
Agronomic traits of the fruits and seeds in WT and *JcUEP:JcGA2ox6* transgenic *Jatropha*. **(A)** Fruits of WT and transgenic *Jatropha* (L43 and 27). **(B,C)** Fruit length and width of WT and transgenic *Jatropha* (L43 and 27). **(D,G)** Seeds of WT and transgenic *Jatropha* (L43 and 27). **(E,F)** Seed length and width of WT and transgenic *Jatropha* (L43 and 27). **(H)** Weight of 10 seeds in WT and transgenic *Jatropha* (L43 and 27). **(I)** Oil content of WT and transgenic *Jatropha* (L43 and 27). The values are presented as the means ± standard deviation. Bars = 1 cm. ^∗∗^Significantly different from the control at the 1% level.

Subsequently, fruit and seed development was affected as well by this transgene. Compared with WT plants, transgenic *Jatropha* lines L43 and L27 exhibited smaller fruits (**Figure 7A**), as the fruit lengths were reduced by 13.04 and 31.59%, respectively (**Figure 7B**), while the widths were almost the same (**Figure 7C**). Consequently, the seeds from the L43 and L27 lines were also small, with 12.90 and 22.04% shorter lengths, respectively, compared with the seed length in WT plants (**Figures 7D–G**). The results indicated that overexpressing *JcGA2ox6* in transgenic *Jatropha* inhibited the elongation of fruits and seeds other than the widths. It is known that GA induces cell elongation (Kende and Zeevaart, 1997). We supposed a reduced GA content caused by overexpression of *JcGA2ox6* would repress cell elongation in fruits and seeds. Consistent with this, in liliaceous *Tricyrtis* sp., most transgenic plants overexpressing *TfGA2ox2* showed no significant differences in cell widths, but the lengths significantly decreased compared with those of control plants (Otani et al., 2013). Furthermore, we analyzed the seed weights and seed oil contents in the L43 and L27 lines. The oil contents were measured using a mini-spec mq-one Seed Analyzer (Bruker Optik, Ettlingen, Germany) (Pan and Xu, 2011). The results showed that the average weights of 10 seeds and the seed oil contents in transgenic lines were significantly decreased (**Figures 7H,I**).

### Effect of Overexpression of *JcGA2ox6* on the Endogenous GA Contents

**FIGURE 8:**
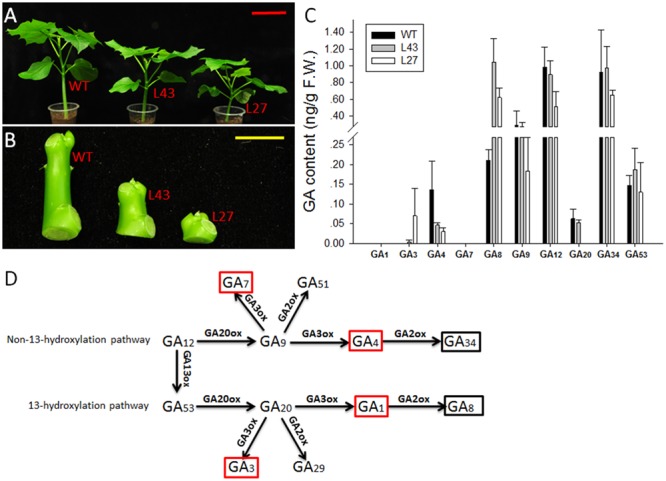
Analysis of GA contents in 6-week-old seedlings from WT and *JcUEP:JcGA2ox6* transgenic *Jatropha* (T1). **(A)** Six-week-old seedlings from WT and transgenic *Jatropha* (L43 and 27). **(B)** Samples of young stems (from shoot tip to node 2) were collected for GA quantification. **(C)** GA contents of WT and transgenic *Jatropha* (L43 and 27). The values are presented as the means ± standard deviation. **(D)** The schematic representation of the principal pathways of GA metabolism in higher plants. Red box, bioactive GA; Black box, inactive GA. Red bar = 10 cm, yellow bar = 1 cm.

To determine whether the endogenous GA contents in transgenic *Jatropha* (L43 and L27) were affected by *JcGA2ox6*, the non-13-hydroxylated GAs (GA_12_, GA_9_, GA_7_, GA_4_ and GA_34_) and the 13-hydroxylated GAs (GA_53_, GA_20_, GA_3_, GA_1_ and GA_8_) (**Figure 8D**) were determined in 6-week-old T1 and WT seedlings (**Figure 8A**). The results (**Figure 8C**) showed that the levels of bioactive GA_4_ were significantly decreased in transgenic lines and were correlated with the degree of dwarfism (**Figure 8A**). However, the levels of GA_34_, the deactivated product of GA_4_, did not differ significantly between the WT and transgenic lines. Contrary to GA_4_, the bioactive GA_3_ levels were increased in transgenic lines. Therefore, we supposed that the reduced GA_4_ levels caused negative feedback control of GA biosynthetic gene expression, resulting in an increase in GA_3_ levels. Consequently, elevated expression levels of *JcGA20ox1, JcGA20ox3, JcGA3ox1, JcGA3ox2* and *JcGA3ox3* were detected in transgenic lines (Supplementary Figure 4). This effect of negative feedback regulation was previously confirmed in other plant species (Cowling et al., 1998; Suo et al., 2012). Another two bioactive GAs, GA_1_ and GA_7_, were not detected in either the WT or transgenic lines. However, GA_8_, the deactivated product of GA_1_ (Yamaguchi, 2008), accumulated in both lines, and there was a significant increase in its levels in the transgenic lines, implying that elevated expression levels of GA biosynthetic genes caused higher GA_1_ levels than WT plants. This result indicated the GA_1_ in *Jatropha* seedlings was completely deactivated by JcGA2ox. Taken together, it suggests that the dwarf phenotype of transgenic *Jatropha* resulted from a decrease in the endogenous bioactive GA_4_. In addition, the transgenic lines displayed decreased levels of GA_20_, GA_12_ and GA_9_, the immediate precursors of GA_1_ and GA_4_ (**Figure 8D**), while GA_53_ did not change significantly.

## Discussion

In the present study, we identified a GA2-oxidase homologue gene in *Jatropha, JcGA2ox6*. The transgenic *Arabidopsis* and *Jatropha* overexpressing *JcGA2ox6* demonstrated the GA-deficient phenotype of dwarfism. By analyzing the contents of the endogenous GAs in transgenic *Jatropha* seedlings, the levels of bioactive GA_4_ significantly decreased in transgenic lines (**Figure 8C**) and were correlated with the degree of dwarfism (**Figure 8A**). However, there was no equivalent increase in the amount of GA_34_, the deactivated product of GA_4_. This result is probably due to a rapid turnover of GA_34_ to its catabolite (Coles et al., 1999; Thomas et al., 1999). Though GA_4_ was downregulated in transgenic *Jatropha*, GA_3_ was upregulated. This may suggest that the non-13-hdroxylation pathway was redirecting synthesis toward the 13-hydroxylation pathway. Thus, instead of conversion of GA_12_ to GA_9_, GA_4_, flow was toward GA_53_, GA_20_, and then GA_3_. In addition, the bioactive GA_1_ and GA_7_ were not detected in either the WT or transgenic lines, suggesting that they did not function as a promoter in the stem elongation of *Jatropha* seedlings, not to mention GA_3_ which was elevated in the transgenic lines. Obviously, GA_4_ is the major bioactive GA that regulates *Jatropha* stem elongation. Similarly, in *Arabidopsis* and hybrid aspen, the GA_4_ plays a pivotal role in controlling shoot elongation (Xu et al., 1997; Cowling et al., 1998; Israelsson et al., 2004). Perhaps GA_1_, GA_3_ and GA_7_ are required in other aspects of *Jatropha* growth and development, as the major bioactive GA is different in the diverse tissue development stages of some plant species. In rice, the GA_4_ levels were undetectable in the uppermost internodes and GA_1_ was the major bioactive GA in vegetative tissues of rice (Zhu et al., 2006). In contrast, an extremely high amount of GA_4_ accumulated in pollen (Kobayashi et al., 1988). Because drastic GA-dependent reactions occur during the reproductive stage, such as rapid elongation of the heading stem and pollen tube, rice may use the most effective GA at this stage and use GA_1_ for ordinary GA-dependent reactions at the vegetative stage (Ueguchi-Tanaka et al., 2007). In *Populus*, the GA_3_ and GA_4_ regulated different glucan hydrolase family 17 genes (*GH17s*) to govern dormancy cycling at the shoot apex (Rinne et al., 2011).

In addition to the dwarf phenotype, transgenic *Arabidopsis* and *Jatropha* overexpressing *JcGA2ox6* also displayed darker green leaves (**Figures 2D, 4C**) and smaller reproductive organs (**Figures 3, 6A–C,F,G, 7**). It indicated the transgene had similar effects on some aspects of *Arabidopsis* and *Jatropha* growth and development, including stem elongation, leaf expansion, and flower and fruit development. However, the effects of the transgene on the flowering time differed between the transgenic *Arabidopsis* and *Jatropha*. Overexpression of *JcGA2ox6* delayed the flowering time in transgenic *Arabidopsis* (**Figures 2A,B**) with no significant difference in the number of rosette leaves. It suggested that the reduced growth rate caused late flowering. In transgenic *Jatropha*, however, the flowering time was not significantly different from that of the WT plant (**Figure 5F**). Similarly, transgenic plants of liliaceous *Tricyrtis* sp. overexpressing the *TfGA2ox* gene from *T. fournieri* exhibited the dwarf phenotype, but no apparent alteration in the flowering time was observed (Otani et al., 2013). However, overexpression of *PtGA2ox* caused early-flowering in the poplar (Zawaski et al., 2011). These findings suggest that the GA regulation of flowering differs between plant species.

In this study, we expected to promote reproductive growth by reducing the endogenous GA content in *Jatropha*. However, transgenic *Jatropha* showed an unexpected phenotype of fewer inflorescences and flowers. Even the seed weights and oil contents were decreased. The possible reason for this result is the usage of a constitutive *JcUEP* promoter, which is active throughout the entire growth phase of the plant. Because the vegetative growth of transgenic *Jatropha* is partially suppressed (**Figure 4**), there is not enough nutrition provided for blossoming and fructification. In rice, when *OsGA2ox1* was constitutively expressed under the direction of the rice actin promoter, transgenic rice showed severe dwarfism but failed to set grain (Sakamoto et al., 2003). In contrast, the expression of *OsGA2ox1* under the control of the promoter of *OsGA3ox2* resulted in a semi-dwarf phenotype that is normal in flowering and grain development (Sakamoto et al., 2003). To guarantee normal vegetative growth, tissue-specific or inducible promoters that are active in the transition from vegetative to reproductive growth in *Jatropha* should be used to drive *JcGA2ox6*. Although the expected phenotype of vigorous reproductive growth was not exhibited in transgenic *Jatropha*, the dwarf trait may allow for dense field plantation and increase the efficiency of fruit collection in *Jatropha* plantations. For example, during close planting in narrow rows, the suitable density for the thick planting of a new variety of semi-dwarf soybean ranged from 500 thousand plants to 600 thousand plants, producing a high yield of 5467.95 kg/hm^2^ (Zheng et al., 2013). In relay intercropped cotton, the light use efficiency and yield were both increased linearly with plant density (Mao et al., 2014). Propagation of transgenic *Jatropha* overexpressing *JcGA2ox6* for dense plantation is in progress.

## Author Contributions

Y-XH, Y-BT, and Z-FX designed the experiments. Y-XH performed the experiments and analyzed the data. Y-XH, Y-BT, and Z-FX wrote the paper.

## Conflict of Interest Statement

The authors declare that the research was conducted in the absence of any commercial or financial relationships that could be construed as a potential conflict of interest.

## Footnotes

1 http://www.ncbi.nlm.nih.gov/BLAST/

2 http://www.megasoftware.net
