# Supplementary material for: Overexpression of Jatropha Gibberellin 2-oxidase 6 (JcGA2ox6) Induces Dwarfism and Smaller Leaves, Flowers and Fruits in Arabidopsis and Jatropha
Source: Front Plant Sci. 2017 Dec 12;8:2103. doi: 10.3389/fpls.2017.02103 (PMC5733080; doi:10.3389/fpls.2017.02103)
Supplement: Supplementary file 1 [file Data_Sheet_1.DOCX]

***Supplementary Material***

**Overexpression of *Jatropha Gibberellin 2-oxidase 6* (*JcGA2ox6*) Induces Dwarfism and Smaller Leaves, Flowers and Fruits in *Arabidopsis* and *Jatropha***

**Ying-Xiong Hu ^1, 2^, Yan-Bin Tao ^1,^** *** and Zeng-Fu Xu ^1,^ ***

*****Author to whom correspondence should be addressed; E-mail: taoyanbin@xtbg.ac.cn, [zfxu@xtbg.ac.cn](mailto:zfxu@xtbg.ac.cn)

# Supplementary Figures and Tables

## Supplementary Figures

**
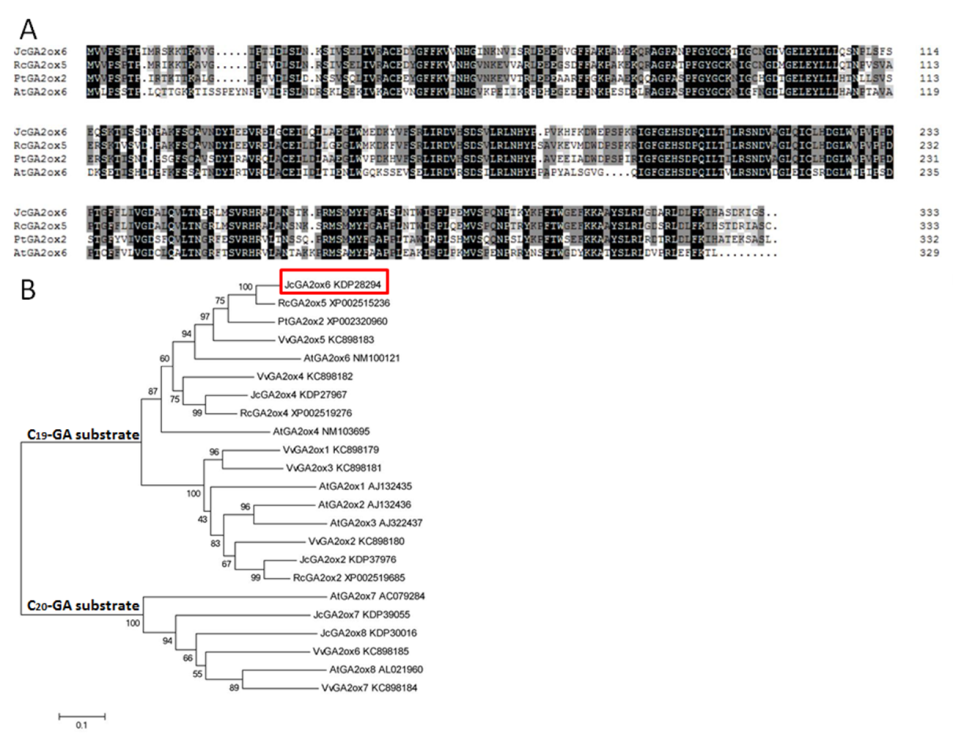
**

**Supplementary Figure 1.** Comparison and phylogenetic analysis of GA2ox homologs. **(A)** Sequence alignment of the JcGA2ox6, RcGA2ox5, PtGA2ox2, and AtGA2ox6 amino acid sequences. Identical amino acid residues are shaded in black, and similar residues are shaded in grey. **(B)** Phylogenetic analysis of GA2ox homologs from different plant species: *Jatropha curcas* JcGA2ox2, JcGA2ox4, JcGA2ox6, JcGA2ox7, JcGA2ox8; *Arabidopsis thaliana* AtGA2ox1, AtGA2ox2, AtGA2ox3, AtGA2ox4, AtGA2ox6, AtGA2ox7, AtGA2ox8; *Vitis vinifera* VvGA2ox1, VvGA2ox2, VvGA2ox3, VvGA2ox4, VvGA2ox5, VvGA2ox6, VvGA2ox7; *Ricinus communis* RcGA2ox2, RcGA2ox4, RcGA2ox5; and *Populus trichocarpa* PtGA2ox2. The phylogeny of these GA2ox homologs was determined based on their amino acid sequences using MEGA5 and the neighbour-joining method. Bootstrap values were obtained using 1,000 bootstrap replicates.

**
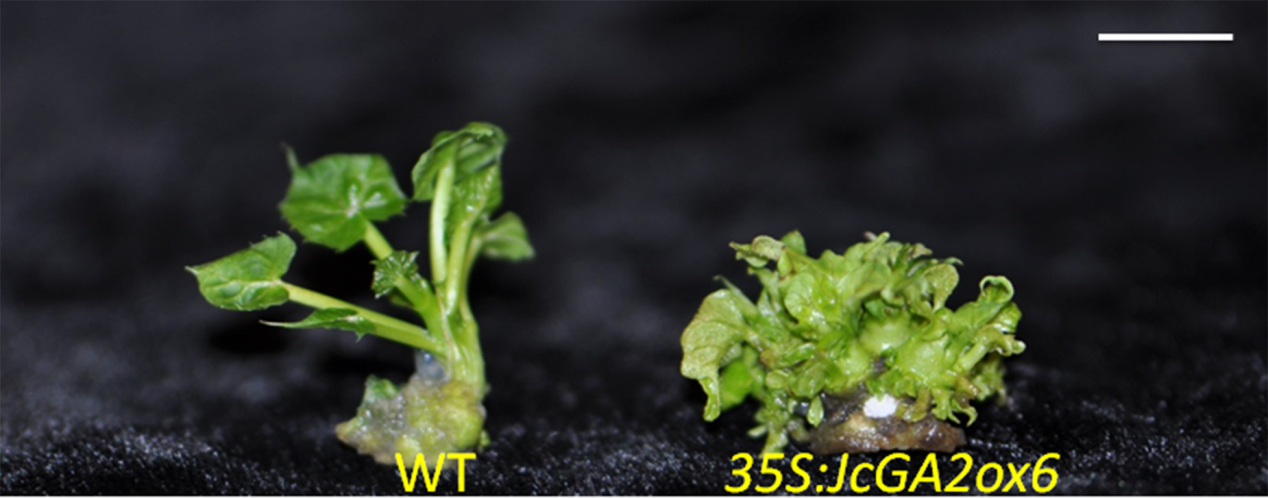
**

**Supplementary Figure 2.** The *35S:JcGA2ox6* transgenic *Jatropha* showed abnormal development of regenerated shoots during the tissue culture period. Bar = 1 cm.


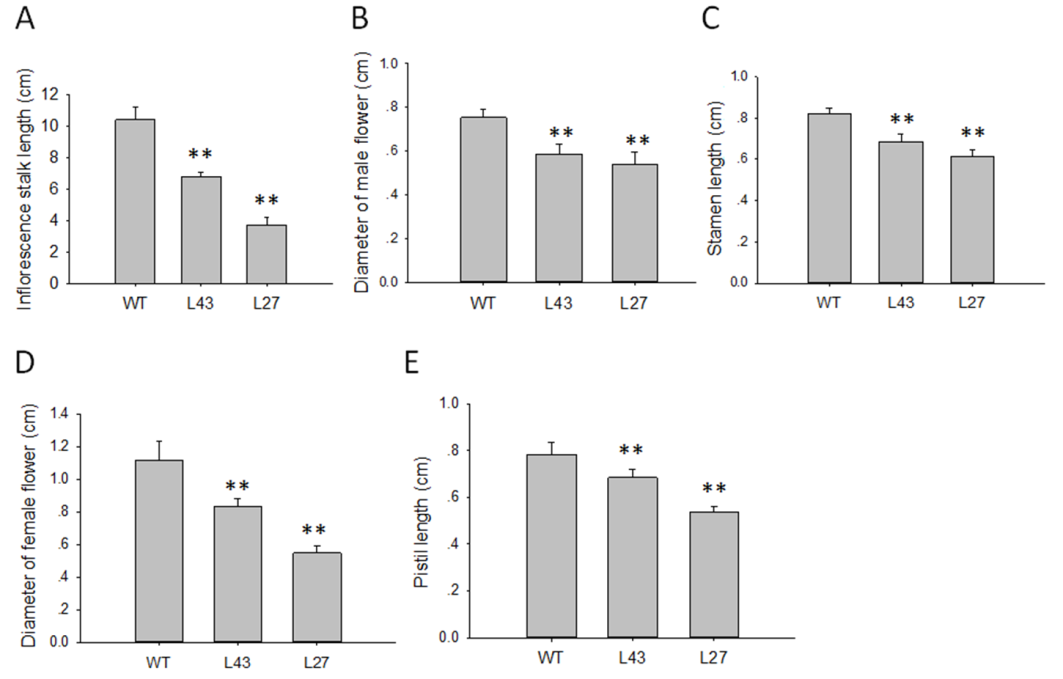


**Supplementary Figure 3.** Phenotypic comparison of inflorescences and flowers in WT and *JcUEP:JcGA2ox6* transgenic *Jatropha* (L43 and 27). **(A)** Inflorescence stalk lengths. **(B)** Male flower diameters. **(C)** Stamen lengths. **(D)** Female flower diameters. **(E)** Pistil lengths. The values are presented as the means ± standard deviation. **significantly different from the control at the 1% level.


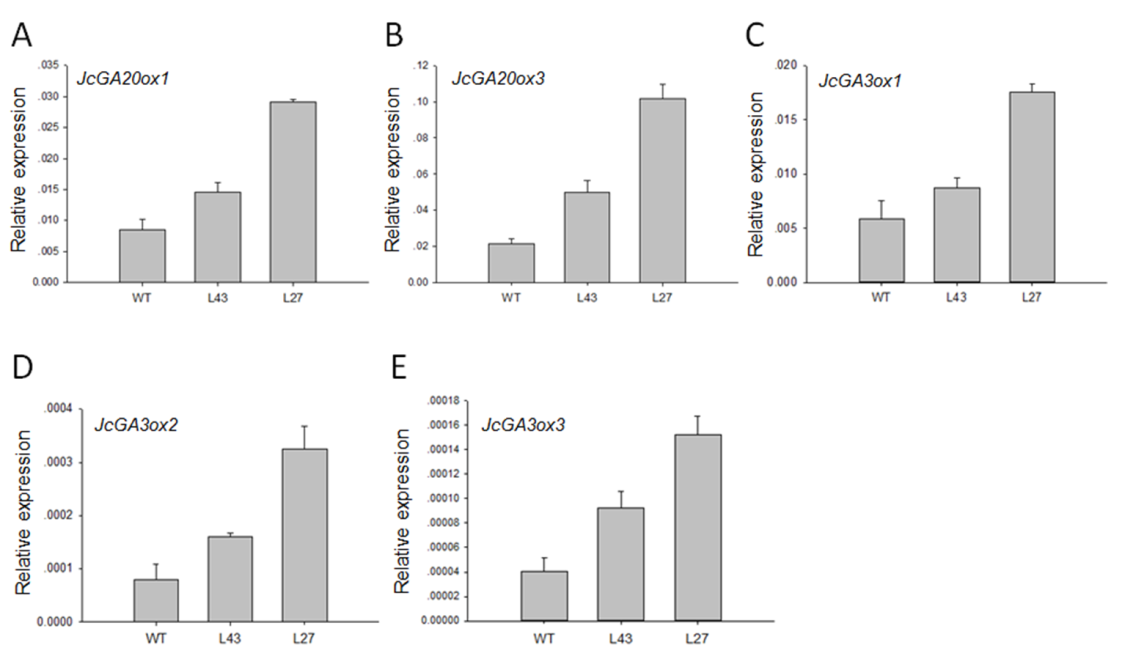


**Supplementary Figure 4.** Expression levels of GA biosynthesis-related genes in WT and *JcUEP:JcGA2ox6* transgenic *Jatropha* (L43 and 27). **(A–E)** Expression levels of *JcGA20ox1* (GenBank accession No. KDP20150), *JcGA20ox3* (KDP22353), *JcGA3ox1* (KDP37169), *JcGA3ox2* (KDP32294), and *JcGA3ox3* (KDP32333) were individually examined. Samples of mature leaves were collected for gene expression analysis. Three independent biological replicates and three technical replicates were measured for each sample. The values are presented as the means ± standard deviation.

## Supplementary Tables

**Supplementary Table 1.** Sequences of the primers used in this study

| **Target template** | **Primer name** | **Primer sequence** |
| --- | --- | --- |
| *JcGA2ox6* full length | XA579-BamHI F  XA580-SalI R | CGGGATCCATGGTAGTTCCGTCTCCAACTCC  GCGTCGACTCAAGACCCGATTTTATCAGAAGC |
| *JcUEP* promoter | XB348-HindIII F  XB349-SacI R | CCCAAGCTTATCTAACATATTATGCG  CGAGCTCGAGAGGAGCAGTAGCCGAA |
| *JcGA2ox6* qRT-PCR | XC251 F  XC252 R | GTCAATCGTGTCTGAACTGATCGTC  ACCAACTCCTTCCTCTTCTAATCTCG |
| *JcGA20ox1* qRT-PCR | XB181 F  XB182 R | TGTCGCTGCAATGGAGGCTTC  GCTGCTGGCATATCCACAGTGT |
| *JcGA20ox3* qRT-PCR | XB856 F  XB857 R | CCAACCGCTAATGCACCAGAAC  ACGCCGTGATTAACGACGAGAA |
| *JcGA3ox1* qRT-PCR | XB546 F  XB547 R | CTCCACTCTACTCACCATTCTCTACC  GACAACAAGCCCGCCAGAAATAG |
| *JcGA3ox2* qRT-PCR | XB548 F  XB549 R | ACCATCCACTCCATCTTCACCAAA  ATCAGGATCGTTCAGGTCAATCGT |
| *JcGA3ox3* qRT-PCR | XC362 F  XC363 R | GCTTGGCTCCTCATACAGATTCAAC  AACCGCTCGATGCAGTACACTT |
| *JcActin1* qRT-PCR | XK191 F  XK192 R | CTCCTCTCAACCCCAAAGCCAA  CACCAGAATCCAGCACGATACCA |
| *AtActin2* qRT-PCR | XK718 F  XK719 R | TGTGCCAATCTACGAGGGTTT  TTTCCCGCTCTGCTGTTGT |
